# Diagnostics Literacy Advocacy Model for Vulnerable Populations

**DOI:** 10.3390/diagnostics12030716

**Published:** 2022-03-15

**Authors:** Tivani P. Mashamba-Thompson

**Affiliations:** Faculty of Health Sciences, University of Pretoria, Pretoria 0002, South Africa; tivani.mashamba-thompson@up.ac.za

**Keywords:** vulnerable populations, literacy, diagnostics, advocacy

## Abstract

Evidence shows that vulnerable populations have lower levels of health literacy, resulting in poor health-seeking behavior and poor uptake of diagnostics. Being health literate promotes health care-seeking behavior and improves engagement with diagnostic services. In this editorial, I define health literacy in the context of access to technology for enabling disease screening, diagnosis and linkage to care. I refer to health literacy in this context as diagnostics literacy. The COVID-19 pandemic has taught us that vulnerable populations are disproportionately disadvantaged by the disruptive measures put in place to control the spread of the virus. Many vulnerable populations are still experiencing short-and longer-term socio-economic consequences. I propose a multi-level diagnostics literacy advocacy model to help improve diagnostic uptake among vulnerable populations.

In response to the COVID-19 pandemic, researchers have scaled up the development of new diagnostics [1]. Effective diagnostics improve detection of SARS-CoV-2 infected patients and the overall surveillance of the COVID-19 pandemic. Recent literature shows that 47% of the world’s population has poor access to diagnostics and are failing to achieve the United Nations General Assembly (UNGA) COVID-19 testing targets of one test per 1000 people per day [2]. Improving access to diagnostics by removing barriers may reduce annual premature deaths by 1.1 million (2.5% of total annual deaths) and morbidity by 38.5 million (1.8% of all conditions) annual disability-adjusted life-years lost in low-income and middle-income countries (LMICs) [3]. Resource-constrained settings with limited laboratory infrastructure also have poor access to diagnostics. Additionally, the SARS-CoV-2 virus spreads inequitably through vulnerable populations with limited access to healthcare, resulting in higher rates of infection and complications. Vulnerable population groups have also been disproportionately disadvantaged by disruptive measures put in place to stop the spread of the virus. Many people who relied on small day-to-day earnings lost income required to meet basic needs such as housing and food. Women and girls who were caring for family members lost educational and professional opportunities and will continue to experience long-term socio-economic consequences. Vulnerable population groups may have lower levels of health literacy, resulting in sub-optimal health-seeking behavior and poor diagnostics uptake.

Improving access to diagnostics is a global health priority and we need to explore factors that limit access and use of available diagnostics in settings that have limited access to laboratory infrastructure. Health literacy is known to influence the use of health services [4]. Health literacy is a major determinant of health outcomes and is imperative to global health [5]. Health literacy is defined as a set of skills that allows patients to control their own well-being, allows them to make smart healthcare choices, improves patients’ communication with healthcare workers and gives them the information to advocate for themselves in healthcare settings [6]. High levels of health literacy improve access to health care services, including diagnostics [7]. Here, I define health literacy in the context of accessing technology to enable disease screening, diagnosis and linkage to care. I refer to health literacy in this context as diagnostics literacy (DL). Diagnostic literacy encompasses a broad range of factors that are closely related to health promotion, including culture, individual empowerment, community development, media and numeracy. We urgently need to implement a multi-level DL advocacy model (Figure 1) to improve diagnostics uptake among underserved populations. 

**The Proposed Multi-Level Diagnostics Literacy Advocacy Model**

**1.**  **Macro level—Advocacy**

- Conduct a key stakeholder workshop and invite all relevant community leaders, policy makers and implementers.
- Establish a disease diagnostics advocacy group comprising diagnostics experts, health promotion experts and health experts.
- Develop and implement tailored disease testing and linkage-to-care advocacy programs for different population groups.

**2.**  **Meso level—Social mobilization**

- Create social media platforms that inform societies about the importance of regular and rapid disease testing, with information about linkage-to-care to remove physical barriers that traditionally impede access to disease testing support and resources.
- Collaborate with religious leaders to develop faith-based diagnostic advocacy programs to help influence health-seeking behaviors and increase engagement with disease testing and linkage-to-care services.
- Develop local newspaper, TV and radio station based diagnostic advocacy programs.

**3.**  **Micro level—Develop knowledge capacity**

- Educate health workers during a training program on disease diagnostics literacy. The curriculum could be delivered using a learning management system linked app.
- Incorporate diagnostic literacy as part of school-based health education services. Edutainment could be used as a public health communication intervention to improve diagnostic literacy through music, jingles, poetry, dramas and puppetry.
- Incorporate diagnostics literacy as part of community-based health education services.

Diagnostic literacy should be improved in vulnerable populations. Well-structured advocacy strategies should be prioritized. Improved DL is likely to lead to increased up take of diagnostics services. Messages can be disseminated using channels tailored to communities to enhance and promote disease diagnosis-seeking behavior. Communities should be fully engaged to ensure that the messages become engrained and translate to health-seeking behavior.

## Figures and Tables

**Figure 1:**
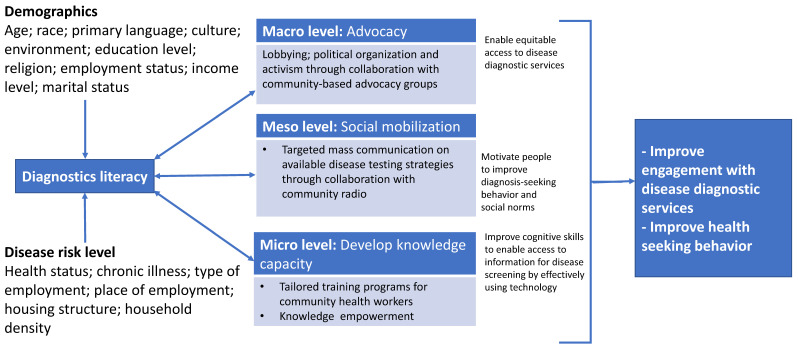
Multi-level diagnostics literacy advocacy model.
